# Surprising Conformers of the Biologically Important A·T DNA Base Pairs: QM/QTAIM Proofs

**DOI:** 10.3389/fchem.2018.00008

**Published:** 2018-02-27

**Authors:** Ol'ha O. Brovarets', Kostiantyn S. Tsiupa, Dmytro M. Hovorun

**Affiliations:** ^1^Department of Molecular and Quantum Biophysics, Institute of Molecular Biology and Genetics, National Academy of Sciences of Ukraine, Kyiv, Ukraine; ^2^Department of Molecular Biotechnology and Bioinformatics, Institute of High Technologies, Taras Shevchenko National University of Kyiv, Kyiv, Ukraine

**Keywords:** A·T DNA base pairs, Watson-Crick, reverse Watson-Crick, Hoogsteen, reverse Hoogsteen, wobble structure, DNA breathing, DNA pre-melting

## Abstract

For the first time novel high-energy conformers–A·T(w_WC_) (5.36), A·T(w_rWC_) (5.97), A·T(w_H_) (5.78), and A·T(w_rH_) (ΔG = 5.82 kcal·mol^−1^) (See **Graphical Abstract**) were revealed for each of the four biologically important A·T DNA base pairs – Watson-Crick A·T(WC), reverse Watson-Crick A·T(rWC), Hoogsteen A·T(H) and reverse Hoogsteen A·T(rH) at the MP2/aug-cc-pVDZ//B3LYP/6-311++G(d,p) level of quantum-mechanical theory in the continuum with ε = 4 under normal conditions. Each of these conformers possesses substantially non-planar wobble (w) structure and is stabilized by the participation of the two anti-parallel N6H/N6H′…O4/O2 and N3H…N6 H-bonds, involving the pyramidalized amino group of the A DNA base as an acceptor and a donor of the H-bonding. The transition states – TS_A·T(WC)↔A·T(wWC)_, TS_A·T(rWC)↔A·T(wrWC)_, TS_A·T(H)↔A·T(wH)_, and TS_A·T(rH)↔A·T(wrH)_, controlling the dipole-active transformations of the conformers from the main plane-symmetric state into the high-energy, significantly non-planar state and *vice versa*, were localized. They also possess wobble structures similarly to the high-energy conformers and are stabilized by the participation of the N6H/N6H′…O4/O2 and N3H…N6 H-bonds. Discovered conformers of the A·T DNA base pairs are dynamically stable short-lived structures [lifetime τ = (1.4–3.9) ps]. Their possible biological significance and future perspectives have been briefly discussed.

## Introduction

Investigation of the dynamics of the isolated DNA base pairs by both the experimental and especially theoretical methods is urgent biophysical task of exceptional importance (Keepers et al., 1982; Pechenaya and Volkov, 1984; Volkov, 1995; Auffinger and Westhof, 1999). At this, the researchers are convinced that exactly the intrinsic conformational dynamics of the DNA base pairs largely determines the functionally important dynamical behavior of DNA and this approach has no reasonable alternatives.

**Graphical Abstract:**
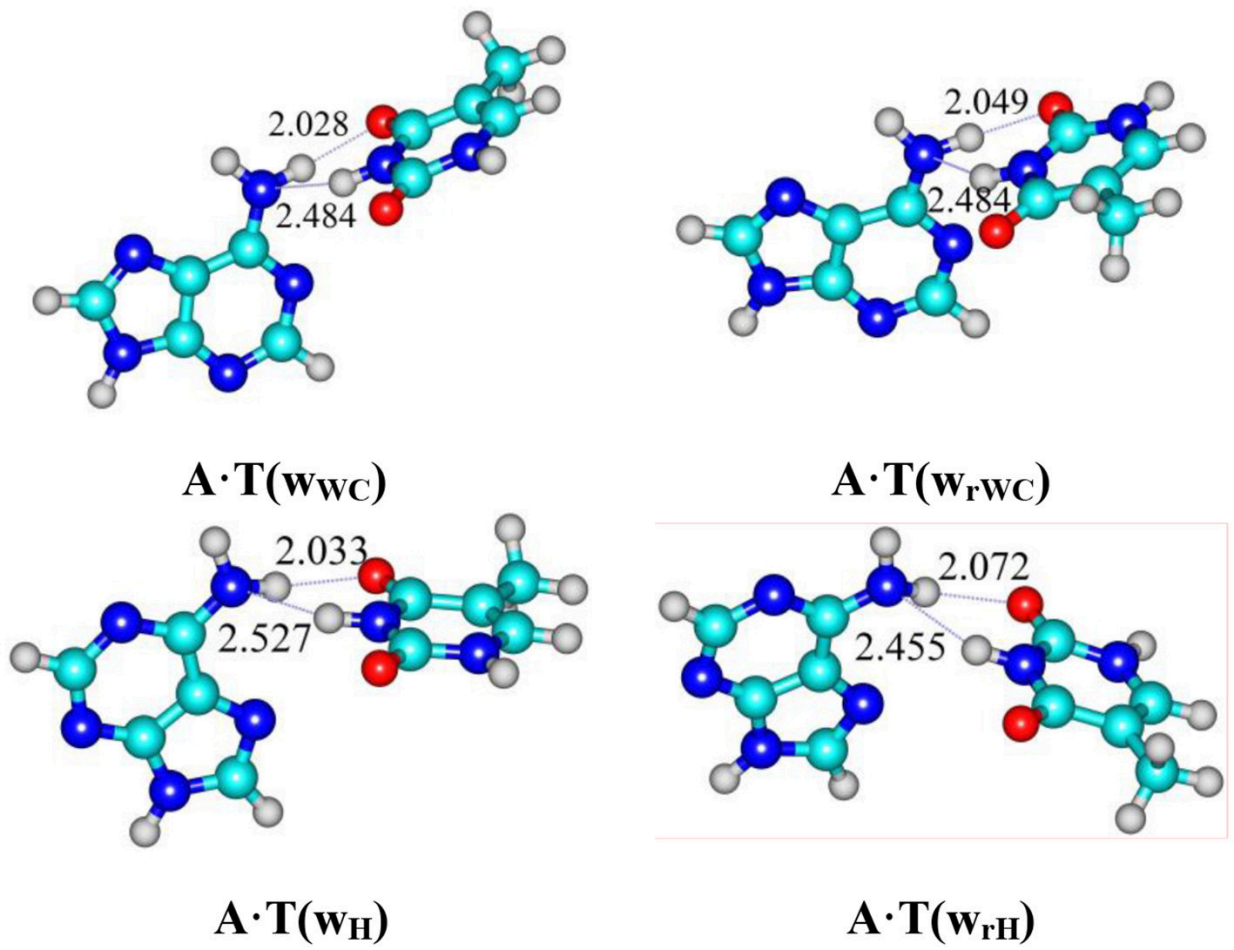
For the first time it was revealed novel high-energy conformers A·T(w_WC_) (5.36), A·T(w_rWC_) (5.97), A·T(w_H_) (5.78) and A·T(w_rH_) (ΔG = 4.98 kcal/mol under normal conditions) at the MP2/aug-cc-pVDZ//B3LYP/6-311++G(d,p) level of quantum-mechanical theory in the continuum with ε = 4.

Spontaneous thermal fluctuations or breathing of DNA enables the opening of the DNA base pairs, making reactive their chemical groups, that are normally hidden inside the DNA double helix, available for hydrogen exchange involving imino and amino groups, chemical modification (e.g., by formaldehyde, that is a toxic, mutagenic and carcinogenic compound leading to fatal consequences or mutagenesis) and important protein-DNA interactions by the participation of the regulatory proteins (Lazurkin et al., 1970; Frank-Kamenetskii and Lazurkin, 1974; Lukashin et al., 1976; Chay, 1979; Frank-Kamenetskii, 1981, 1983, 1985; Guéron et al., 1987; Guéron and Leroy, 1995; von Hippel et al., 2013; Frank-Kamenetskii and Prakash, 2014). Notably, that reactions of the hydrogen exchange and formaldehyde interaction with DNA were the first documented cases evidencing the opening of the DNA base pairs (Lazurkin et al., 1970; Frank-Kamenetskii and Lazurkin, 1974; Lukashin et al., 1976; Chay, 1979; Guéron et al., 1987).

Moreover, it is believed that opening of the DNA base pairs with a defined probability ~10^−5^ (Lazurkin et al., 1970; Frank-Kamenetskii and Lazurkin, 1974; Lukashin et al., 1976; Frank-Kamenetskii, 1981, 1985; Guéron et al., 1987; Guéron and Leroy, 1995; von Hippel et al., 2013; Frank-Kamenetskii and Prakash, 2014) precedes the melting of DNA, that is represent the two-state model according to which each base pair is suggested to stay in the closed or open states (Frank-Kamenetskii and Lazurkin, 1974; Lukashin et al., 1976; Chay, 1979; Frank-Kamenetskii, 1981, 1983; Guéron et al., 1987; Singh and Singh, 2017). Exactly this model could quantitatively explain in details the melting of DNA processing in the multistate way due the different length and heterogeneous sequence (Vologodskii et al., 1984; Wartell and Benight, 1985; Wada and Suyama, 1986; SantaLucia, 1998). At this, predicted lifetimes of the open state of the base pairs lie in the sub microsecond range (~10^−7^ s) (Craig et al., 1971; Porschke and Eigen, 1971; Frank-Kamenetskii, 2011). However, according to the literature data it remains unknown, what the nature of the open state of the DNA base pairs is and whether there is a barrier on the potential energy surface for providing its existence (Lavery, 1994; Stofer et al., 1999; Yang et al., 2015).

It was also demonstrated by NMR experiment (Nikolova et al., 2011, 2013) a Hoogsteen breathing consisting in the flipping of the Watson-Crick DNA base pair from the usual *anti*-conformation to the less favorable *syn*-conformation with probability ~10^−2^, representing another pathway for the reaction of formaldehyde attack on DNA (Bohnuud et al., 2012).

Since the model of two states—H-bonded base pair and opened base pair—is not able to describe in details the dynamical behavior of DNA, which experimentally manifests itself in a number of its physico-chemical properties (Lazurkin et al., 1970; Craig et al., 1971; Porschke and Eigen, 1971; Frank-Kamenetskii and Lazurkin, 1974; Lukashin et al., 1976; Chay, 1979; Frank-Kamenetskii, 1981, 1983, 1985, 2011; Vologodskii et al., 1984; Wartell and Benight, 1985; Wada and Suyama, 1986; Guéron et al., 1987; Lavery, 1994; Guéron and Leroy, 1995; SantaLucia, 1998; Stofer et al., 1999; Giudice et al., 2001; Ababneh et al., 2003; Coman and Russu, 2005; Nikolova et al., 2011, 2013; Bohnuud et al., 2012; von Hippel et al., 2013; Frank-Kamenetskii and Prakash, 2014; Yang et al., 2015; Singh and Singh, 2017), the searching of new conformational states of the DNA base pairs near their Watson-Crick global minima has been intensified (Keepers et al., 1982; Pechenaya and Volkov, 1984; Volkov, 1995; Giudice et al., 2003; Pérez et al., 2007; Lindahl et al., 2017).

The modeling of the conformational heterogeneity of the Watson-Crick A·T DNA base pair allowing the existence of the semiopen states in DNA, which is associated with the presence of the weak C2H…O2 H-bond in it, and their support by the semi-empirical quantum-chemical MNDO/H (Hovorun, 1997) and PM3 (Kryachko and Volkov, 2001) methods presented in the papers (Hovorun, 1997; Kryachko and Volkov, 2001) seems attractive. Moreover, none of these interesting ideas has been confirmed by *ab initio* methods.

Nowadays in the literature it does not present the data confirming the presence of the stable conformational states in the isolated Watson-Crick DNA base pairs, except canonical ones (Lavery, 1994; Stofer et al., 1999). It is obviously connected with the lack of the new ideas according as the structural features of the complementary foundations, so the nature of the intermolecular interactions, first of all of the H-bonds responsible for the presence of the conformers, which differs from the classical ones.

In present work basing on our previous data (Brovarets' and Hovorun, 2014a,b,c, 2015a,b; Glushenkov and Hovorun, 2016), we adhere to the idea that the pyramidalized amino group of the adenine (A) DNA base can simultaneously form two antiparallel N6H/N6H′…O4/O2 and N3H…N6 H-bonds with thymine (T) DNA base, thus supporting high-energy non-planar conformers of the biologically important A·T DNA base pairs. We succeeded to localize the transition states (TSs) connecting the main plane-symmetrical conformers of the A·T base pairs (global minimum) with the established significantly non-planar high-energy conformers. On the basis of the obtained data, we expressed the assumption according the possible biological importance of the discovered conformers of the canonical A·T DNA base pairs.

We chose as the object of the investigation of the biologically-important A·T DNA base pairs, in particular–Watson-Crick A·T(WC), reverse Watson-Crick A·T(rWC), Hoogsteen A·T(H) and reverse Hoogsteen A·T(rH) base pairs (Donohue and Trueblood, 1960; Haschemeyer and Sobell, 1963; Hoogsteen, 1963; Tchurikov et al., 1989; Liu et al., 1993; Parvathy et al., 2002; Sühnel, 2002; Zagryadskaya et al., 2003; Brovarets', 2013a,b; Alvey et al., 2014; Nikolova et al., 2014; Yang et al., 2015; Poltev et al., 2016; Zhou, 2016; Sathyamoorthy et al., 2017; Szabat and Kierzek, 2017; Ye et al., 2017).

Thus, the reverse A·T(rWC) Watson-Crick or so-called Donohue DNA base pair (Donohue and Trueblood, 1960), which is formed by the rotation of one of the bases according to the other by 180° around the N1–N3 axis of the Watson-Crick A·T(WC) DNA base pair, has been registered in the bioactive parallel-stranded DNA (Tchurikov et al., 1989; Parvathy et al., 2002; Brovarets', 2013a,b; Poltev et al., 2016; Szabat and Kierzek, 2017; Ye et al., 2017).

The A·T(H) Hoogsteen base pair (Hoogsteen, 1963) is formed due to the rotation on 180° of the A DNA base relative to the T DNA base around the C9-N9 axis from the *anti* (WC) to *syn* (H) conformation, representing itself alternative DNA conformation that is involved into a number of biologically important processes such as recognition, damage induction, replication and has been actively investigated in the literature (Hoogsteen, 1963; Brovarets', 2013a,b; Alvey et al., 2014; Nikolova et al., 2014; Yang et al., 2015; Zhou, 2016; Sathyamoorthy et al., 2017). In particular, in the canonical DNA double helix Watson–Crick base pairs exist in a dynamic equilibrium with sparsely populated (~0.02–0.4%) and short-lived (lifetimes ~0.2–2.5 ms) Hoogsteen base pairs (Zhou, 2016).

At this, the reverse A·T(rH) Hoogsteen or so-called Haschemeyer–Sobell base pair (Haschemeyer and Sobell, 1963), that is formed by the rotation of one of the bases by 180° around the N7–N3 axis of the base pair according the other base (Brovarets', 2013a,b), also plays important biological role (Liu et al., 1993; Sühnel, 2002; Zagryadskaya et al., 2003).

### Computational methods

#### Density functional theory calculations of the geometry and vibrational frequencies

Geometries of the main and high-energy conformers and transition states (TSs) of their mutual conformational transformations, as well as their harmonic vibrational frequencies have been calculated at the B3LYP/6-311++G(d,p) level of theory (Hariharan and Pople, 1973; Krishnan et al., 1980; Lee et al., 1988; Parr and Yang, 1989; Tirado-Rives and Jorgensen, 2008), using Gaussian'09 package (Frisch et al., 2010). Applied level of theory has proved itself successful for the calculations of the similar systems (Brovarets' and Hovorun, 2010a,b, 2015c; Matta, 2010; Brovarets' et al., 2015b). A scaling factor that is equal to 0.9668 has been applied in the present work for the correction of the harmonic frequencies of all conformers and TSs of their conformational transitions (Palafox, 2014; Brovarets' and Hovorun, 2015c; Brovarets' et al., 2015b; El-Sayed et al., 2015). We have confirmed the local minima and TSs, localized by Synchronous Transit-guided Quasi-Newton method (Peng et al., 1996), on the potential energy landscape by the absence or presence, respectively, of the imaginary frequency in the vibrational spectra of the complexes. We applied standard TS theory for the estimation of the activation barriers of the tautomerisation reaction (Atkins, 1998).

All calculations have been carried in the continuum with ε = 4, that adequately reflects the processes occurring in real biological systems without deprivation of the structurally functional properties of the bases in the composition of DNA and satisfactorily models the substantially hydrophobic recognition pocket of the DNA-polymerase machinery as a part of the replisome (Bayley, 1951; Dewar and Storch, 1985; Petrushka et al., 1986; García-Moreno et al., 1997; Mertz and Krishtalik, 2000; Brovarets' and Hovorun, 2014d,e).

#### Single point energy calculations

We continued geometry optimizations with electronic energy calculations at the single point at the MP2/aug-cc-pVDZ level of theory (Frisch et al., 1990; Kendall et al., 1992).

The Gibbs free energy G for all structures was obtained in the following way:

(1) $$\text{G}={\text{E}}_{\text{el}}{+\text{E}}_{\text{corr}},$$

where E_el_-electronic energy, while E_corr_-thermal correction.

#### Evaluation of the interaction energies

Electronic interaction energies ΔE_int_ have been calculated at the MP2/6-311++G(2df,pd) level of theory as the difference between the total energy of the base pair and energies of the monomers and corrected for the basis set superposition error (BSSE) (Boys and Bernardi, 1970; Gutowski et al., 1986) through the counterpoise procedure (Sordo et al., 1988; Sordo, 2001).

#### Estimation of the kinetic parameters

The time τ_99.9%_ necessary to reach 99.9% of the equilibrium concentration of the reactant and product in the system of the reversible first-order forward (*k*_*f*_) and reverse (*k*_*r*_) reactions was estimated by the formula (Atkins, 1998):

(2) $$\tau_{99.9\%}=\frac{ln10^{3}}{k_{f}+k_{r}}.$$

The lifetime τ of the conformers has been calculated using the formula 1/*k*_*r*_, where the values of the forward *k*_*f*_ and reverse *k*_*r*_ rate constants for the tautomerisation reactions were obtained as (Atkins, 1998):

(3) $$k_{f,r}=\Gamma \cdot \frac{k_{B}T}{h}e^{-\frac{\Delta \Delta G_{f,r}}{RT}},$$

where quantum tunneling effect has been accounted by Wigner's tunneling correction (Wigner, 1932), successfully used for the double proton reactions in DNA base pairs (Brovarets' and Hovorun, 2013, 2014c):

(4) $$\Gamma =1+\frac{1}{24}{\left(\frac{h\nu_{i}}{k_{B}T}\right)}^{2},$$

where *k*_*B*_–Boltzmann's constant, *h*–Planck's constant, ΔΔ*G*_*f,r*_–Gibbs free energy of activation for the conformational transition in the forward (*f*) and reverse (*r*) directions, ν_*i*_–magnitude of the imaginary frequency associated with the vibrational mode at the TSs.

#### QTAIM analysis

Bader's quantum theory of Atoms in Molecules (QTAIM) (Bader, 1990; Matta and Hernández-Trujillo, 2003; Matta et al., 2006a; Cukrowski and Matta, 2010; Matta, 2014; Lecomte et al., 2015), using program package AIMAll (Keith, 2010), was applied to analyse the electron density distribution. The presence of the bond critical point (BCP), namely the so-called (3,−1) BCP, and a bond path between hydrogen donor and acceptor, as well as the positive value of the Laplacian at this BCP (Δρ > 0), were considered as criteria for the H-bond formation (Bader, 1990; Matta and Hernández-Trujillo, 2003; Matta et al., 2006a; Cukrowski and Matta, 2010; Matta, 2014; Lecomte et al., 2015). Wave functions were obtained at the level of theory used for geometry optimisation.

#### Calculation of the energies of the intermolecular H-bonds

The energies of the intermolecular uncommon H-bonds (Brovarets' et al., 2013, 2015a) in the base pairs were calculated by the empirical Espinosa-Molins-Lecomte (EML) formula based on the electron density distribution at the (3,−1) BCPs of the specific contacts (Espinosa et al., 1998; Matta, 2006; Matta et al., 2006b; Mata et al., 2011; Brovarets' et al., 2014a):

(5) E=0.5·V(r),

where V(r) – value of a local potential energy at the (3,−1) BCP.

The energies of all other conventional AH···B H-bonds were evaluated by the empirical Iogansen's formula (Iogansen, 1999):

(6) $$E_{AH\;\cdot \cdot \cdot B}=0.33\cdot \sqrt{\Delta \nu -40},$$

where Δν—magnitude of the frequency shift of the stretching mode of the AH H-bonded group involved in the AH···B H-bond relatively the unbound group. The partial deuteration was applied to minimize the effect of vibrational resonances (Brovarets' and Pérez-Sánchez, 2016a, 2017; Brovarets' et al., 2016, 2017a,b, 2018; Brovarets' and Hovorun, in press).

The atomic numbering scheme for the DNA bases is conventional (Saenger, 1984).

## Results and their discussion

**Figure 1:**
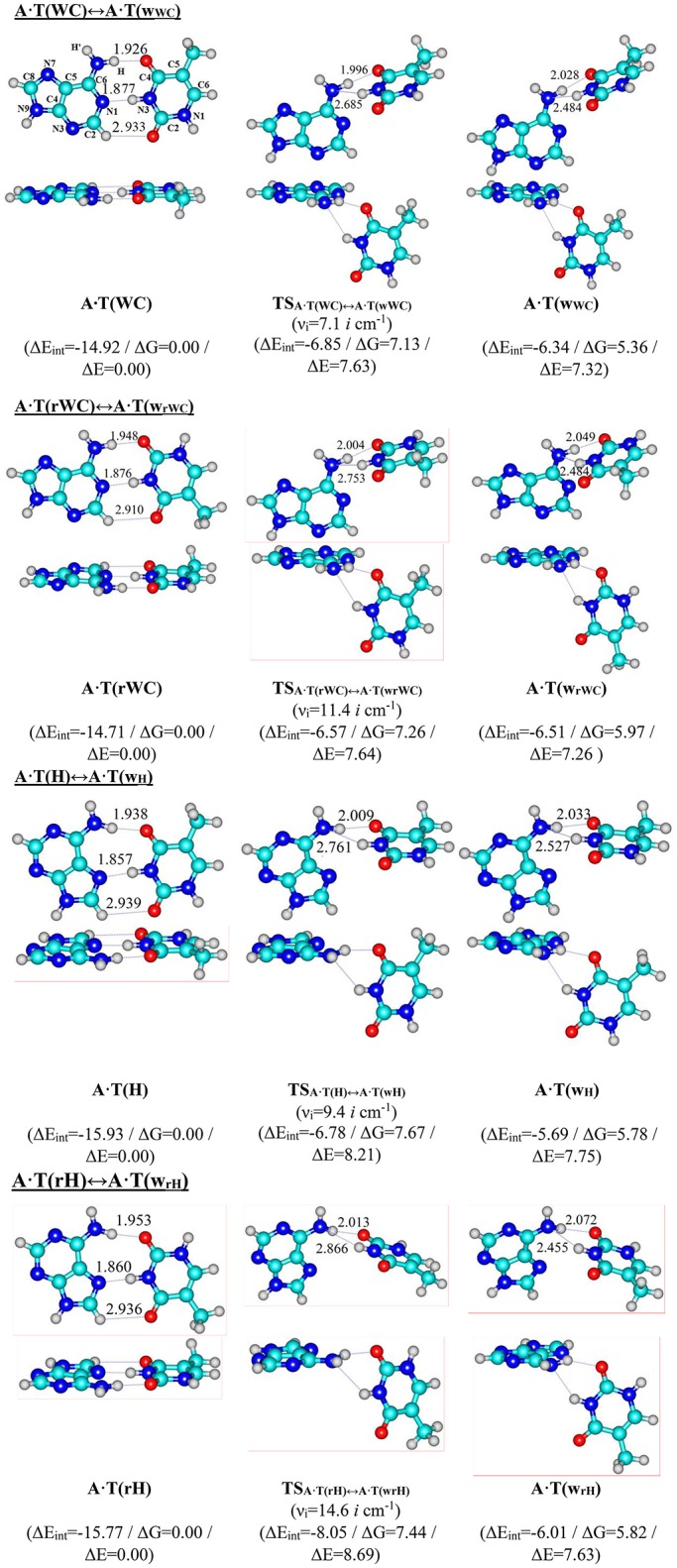
Reaction pathways of the discovered conformational transitions of the four biologically important A·T DNA base pairs. Electronic energies of the interaction ΔE_int_ (MP2/6-311++G(2df,pd)//B3LYP/6-311++G(d,p) level of theory, in kcal·mol^−1^), relative Gibbs free energies ΔG and electronic energies ΔE (in kcal·mol^−1^), imaginary frequencies ν_*i*_ at the TSs of the conformational transitions (MP2/aug-cc-pVDZ//B3LYP/6-311++G(d,p) level of theory in the continuum with ε = 4 at *T* = 298.15 K) are presented below pathways in brackets. Dotted lines indicate AH···B H-bonds – their lengths are presented in angstroms (for more detailed physico-chemical characteristics of the H-bonds see Table 2); carbon atoms are in light-blue, nitrogen – in dark-blue, hydrogen – in gray and oxygen – in red.

For the first time we have detected on the potential (electronic) energy surface of each of the four biologically important A·T(WC), A·T(rWC), A·T(H) and A·T(rH) DNA base pairs the shallow local minima (ΔΔE < kT under normal conditions) corresponding to the dynamically stable A·T(w_WC_), A·T(w_rWC_), A·T(w_H_) and A·T(w_rH_) conformers, correspondingly, with shifted, wobble (w) architecture (Figure 1). These conformers possess significantly non-planar structure (see Table 1 with the selected angles of the non-planarity) and C_1_ point group of symmetry. At this, the piramidalized amino group of the A DNA base is involved into the intermolecular H-bonding with T base through two anti-parallel N6H…O4/O2 and N3H…N6 H-bonds in the A·T(WC)/A·T(rWC) base pairs and N6H′…O4/O2 and N3H…N6 H-bonds in the A·T(H)/A·T(rH) DNA base pairs. In all conformers and TSs without exception the N3H…N6 H-bonds with significantly increased ellipticity are weaker than the N6H/N6H′…O4/O2 H-bonds (Table 2). These interactions should be attributed to the weak and medium H-bonds according to the existing classification (Saenger, 1984). Their most important characteristics are presented in Table 2. It should be noted that each of the four investigated A·T DNA base pairs in the basic plane-symmetric conformation is stabilized by the participation of the three intermolecular H-bonds, one of which, namely, the C2H/C8H…O4/O2 is non-canonical (Brovarets' et al., 2013, 2015a). For all A·T DNA base pairs without exception the middle N3H…N1/N7 H-bonds are the strongest (~7 kcal·mol^−1^). At this, the total energy of the intermolecular H-bonds in each complex consists only some part of the total electronic energy of the interaction between the bases (Figure 1, Table 2). The same regularity is observed for the other DNA base pairs (Brovarets' et al., 2014b; Brovarets' and Hovorun, 2015d,e,f,g, 2016b). For all conformers without exception the amino H or H' atom of the A DNA base, that directly takes part in the H-bonding with T DNA base, significantly deviates from the plane of the purine ring in comparison with the other H′ or H hydrogen atom (Table 1). In all cases the high-energy conformers of the biologically important A·T base pairs are more polar than main conformers (Table 2).

**Table 1:** Selected geometrical parameters characterizing the non-planarity of the discovered conformers of the four biologically important A·T DNA base pairs and TSs of their conformational transitions to the main conformers with plane symmetry, obtained at the B3LYP/6-311++G(d,p) level of theory in the continuum with ε = 4.

**Complex/Base**	**Dihedral angle, degree**		
	**C5C6N6H′**	**N1C6N6H**	**HN9N1H**
A·T(w_WC_)	−13.8	14.9	−44.4
TS_A·T(WC)↔A·T(wWC)_	−11.5	12.7	−44.1
A·T(w_rWC_)	−14.2	15.4	99.4
TS_A·T(rWC)↔A·T(wrWC)_	−11.1	12.1	98.8
A·T(w_H_)	16.8	−12.9	−25.0
TS_A·T(H)↔A·T(wH)_	13.7	−10.2	−24.9
A·T(w_rH_)	18.2	−14.0	88.0
TS_A·T(rH)↔A·T(wrH)_	12.6	−10.1	84.4
A	−7.2	6.6	–

**Table 2:** Electron-topological, geometrical and energetic characteristics of the intermolecular H-bonds in the investigated conformers of the A·T DNA base pairs and TSs of their conformational transformations obtained at the B3LYP/6-311++G(d,p) level of theory (ε = 4) (see Figure 1).

**Complex**	**AH···B H-bond**	**ρ^a^**	****Δρ**^b^**	***100·ε*^c^**	***d_*A*…*B*_*^d^**	***d_*H*…*B*_*^e^**	**AH…B^f^**	***E_*AH*…*B*_*^g^**	**μ^h^**
A·T(WC)	N6H…O4	0.026	0.092	4.69	2.945	1.928	174.6	4.70	2.47
	N3H…N1	0.036	0.090	6.74	2.919	1.878	178.4	7.22	
	C2H…O2	0.004	0.013	3.31	3.746	2.932	131.9	0.68^*^	
TS_A·T(WC)↔A·T(wWC)_	N6H…O4	0.023	0.081	1.95	2.981	1.996	162.2	4.19	3.98
	N3H…N6	0.007	0.021	82.90	3.498	2.685	137.2	2.63	
A·T(w_WC_)	N6H…O4	0.022	0.076	2.10	2.988	2.028	156.2	4.11	3.97
	N3H…N6	0.010	0.030	31.69	3.337	2.484	141.1	1.75	
A·T(rWC)	N6H…O2	0.024	0.089	5.53	2.964	1.948	174.1	4.95	3.34
	N3H…N1	0.036	0.090	6.74	2.917	1.876	177.6	7.22	
	C2H…O4	0.004	0.014	3.16	3.728	2.910	132.3	0.72^*^	
TS_A·T(rWC)↔A·T(wrWC)_	N6H…O2	0.022	0.079	2.05	2.989	2.004	168.3	4.18	3.88
	N3H…N6	0.006	0.019	97.29	3.546	2.753	135.2	2.35	
A·T(w_rWC_)	N6H…O2	0.020	0.072	1.98	3.000	2.049	154.6	3.85	3.71
	N3H…N6	0.010	0.030	26.08	3.332	2.484	140.6	1.81	
A·T(H)	N6H′…O4	0.025	0.091	4.04	2.944	1.938	169.0	4.46	7.91
	N3H…N7	0.037	0.095	6.07	2.895	1.857	177.0	7.03	
	C8H…O2	0.004	0.014	10.61	3.607	2.939	120.3	0.68^*^	
TS_A·T(H)↔A·T(wH)_	N6H′…O4	0.022	0.079	1.90	2.979	2.009	158.7	4.00	8.57
	N3H…N6	0.006	0.019	138.56	3.562	2.761	136.0	2.53	
A·T(w_H_)	N6H′…O4	0.021	0.075	2.64	2.983	2.033	154.4	4.01	8.29
	N3H…N6	0.009	0.028	34.33	3.370	2.527	140.1	1.55	
A·T(rH)	N6H′…O2	0.024	0.089	4.98	2.958	1.953	169.3	4.21	7.14
	N3H…N7	0.037	0.094	6.12	2.899	1.860	178.2	6.96	
	C8H…O4	0.004	0.014	10.97	3.605	2.936	120.4	0.68^*^	
TS_A·T(rH)↔A·T(wrH)_	N6H′…O2	0.022	0.079	1.17	2.983	2.012	159.0	3.78	8.75
	N3H…N6	0.006	0.018	139.56	3.639	2.866	133.5	2.57	
A·T(w_rH_)	N6H′…O2	0.020	0.069	2.88	2.998	2.072	150.5	3.71	8.26
	N3H…N6	0.010	0.032	21.42	3.308	2.455	141.1	1.55	

a *The electron density at the (3,−1) BCP of the H-bond, a.u*.

b *The Laplacian of the electron density at the (3,−1) BCP of the H-bond, a.u*.

c *The ellipticity at the (3,−1) BCP of the H-bond*.

d *The distance between the A (H-bond donor) and B (H-bond acceptor) atoms of the AH…B H-bond, Å*.

e *The distance between the H and B atoms of the AH…B H-bond, Å*.

f *The H-bond angle, degree*.

g *The energy of the H-bonds, calculated by Iogansen's or Espinose-Molins-Lecomte (marked with an asterisk) formulas, kcal·mol^−1^*.

h *The dipole moment of the complex, D*.

We have also localized the non-planar transition states of the A·T(WC)↔A·T(w_WC_), A·T(rWC)↔A·T(w_rWC_), A·T(H)↔A·T(w_H_) and A·T(rH)↔A·T(w_rH_) conformational transitions - TS_A·*T*(*WC*)↔*A*·*T*(*wWC*)_, TS_A·*T*(*rWC*)↔*A*·*T*(*wrWC*)_, TS_A·*T*(*H*)↔*A*·*T*(*wH*)_ and TS_A·*T*(*rH*)↔*A*·*T*(*wrH*)_, respectively, with low values of imaginary frequency (7.1, 11.4, 9.4 and 14.6 *i* cm^−1^). These wobble structures (Table 1) are supported by the couple of the anti-parallel intermolecular H-bonds - N6H…O4/O2 and N3H…N6 H-bonds (A·T(WC)↔A·T(w_WC_) and A·T(rWC)↔A·T(w_rWC_), respectively), N6H′…O4/O2 and N3H…N6 H-bonds (A·T(H)↔A·T(w_H_) and A·T(rH)↔A·T(w_rH_), respectively) (Figure 1, Table 2). Characteristically, that all revealed conformational transitions without exception are dipole-active, since they are accompanied by the changing of the dipole moment of the initial and terminal base pairs. At this, TSs of each conformational transition have maximal value of the dipole moment (Table 2).

Main characteristics of the investigated conformational transitions are presented in Table 3. Analysis of these data points that short-lived conformers are dynamically-stable structures with the lifetimes (1.4–3.9) · 10^−12^ s. Really, for all of them the energy of zero vibrations, which frequency become imaginary in the TS, is less than the electronic energy of the electronic energy barrier ΔΔE for the reverse conformational transition and Gibbs free energy barrier for the reverse conformational transition ΔΔG > 0 under normal conditions. Notably, the range of the six low-frequency intermolecular vibrations of the discovered conformers is significantly shifted to the low-frequency region comparably with the main conformational states. These data points on the fact that revealed conformers are quite soft structures, that could be easily deformed under the influence of the external forces, in particular, caused by the stacking interactions with the neighboring DNA bases.

**Table 3:** Energetic and kinetic characteristics of the discovered conformational transitions of the four biologically important A·T DNA base pairs obtained at the MP2/6-311++G(2df,pd)//B3LYP/6-311++G(d,p) (marked by the asterisk) and MP2/aug-cc-pVDZ//B3LYP/6-311++G(d,p) (marked by the double asterisk) levels of theory in the continuum with ε = 4.

**Conformational transition**	ν**_i_^a^**	Δ**G^b^**	Δ**E^c^**	Δ**ΔG_TS_^d^**	Δ**ΔE_TS_^e^**	Δ**ΔG^f^**	Δ**ΔE^g^**	**k_f_^h^**	**k_r_^i^**	τ_99.9%_**^j^**	τ**^k^**
A·T(WC)↔A·T(w_WC_)*	7.1	4.45	6.41	6.13	6.63	1.68	0.22	1.98·10^8^	3.62·10^11^	1.91·10^−11^	2.76·10^−12^
A·T(WC)↔A·T(w_WC_)**	7.1	5.36	7.32	7.13	7.63	1.77	0.31	3.64·10^7^	3.11·10^11^	2.22·10^−11^	3.22·10^−12^
A·T(rWC)↔A·T(w_rWC_)*	11.4	5.06	6.35	6.26	6.64	1.20	0.29	1.60·10^8^	8.16·10^11^	8.47·10^−12^	1.23·10^−12^
A·T(rWC)↔A·T(w_rWC_)**	11.4	5.97	7.26	7.26	7.64	1.29	0.38	2.95·10^7^	7.03·10^11^	9.83·10^−12^	1.42·10^−12^
A·T(H)↔A·T(w_H_)*	9.4	4.98	6.94	6.76	7.29	1.78	0.35	6.85·10^7^	3.06·10^11^	2.26·10^−11^	3.27·10^−12^
A·T(H)↔A·T(w_H_)**	9.4	5.78	7.75	7.67	8.21	1.89	0.45	1.46·10^7^	2.55·10^11^	2.71·10^−11^	3.92·10^−12^
A·T(rH)↔A·T(w_rH_)*	14.6	5.04	6.85	6.46	7.72	1.42	0.87	1.13·10^8^	5.61·10^11^	1.23·10^−11^	1.78·10^−12^
A·T(rH)↔A·T(w_rH_)**	14.6	5.82	7.63	7.44	8.69	1.62	1.07	2.16·10^7^	4.01·10^11^	1.72·10^−11^	2.49·10^−12^

a *The imaginary frequency at the TS of the conformational transition, cm^−1^*.

b *The Gibbs free energy of the product relatively the reactant of the conformational transition (T = 298.15 K), kcal·mol^−1^*.

c *The electronic energy of the product relatively the reactant of the conformational transition, kcal·mol^−1^*.

d *The Gibbs free energy barrier for the forward conformational transition, kcal·mol^−1^*.

e *The electronic energy barrier for the forward conformational transition, kcal·mol^−1^*.

f *The Gibbs free energy barrier for the reverse conformational transition, kcal·mol^−1^*.

g *The electronic energy barrier for the reverse conformational transition, kcal·mol^−1^*.

h *The forward rate constant for the conformational transition, s^−1^*.

I *The reverse rate constant for the conformational transition, s^−1^*.

j *The time necessary to reach 99.9% of the equilibrium concentration between the reactant and the product of the conformational transition, s*.

k *The lifetime of the product of the conformational transition, s*.

The methyl group of the T DNA base does not change its orientation during the process of the conformational transformations. Moreover, the heterocycles of the bases remain planar, despite their ability for the out-of-plane bending (Govorun et al., 1992; Hovorun et al., 1999; Nikolaienko et al., 2011).

Special attention should be payed to the characteristic specificities of the A·T(WC)↔A·T(w_WC_), A·T(rWC)↔A·T(w_rWC_), A·T(H)↔A·T(w_H_) and A·T(rH)↔A·T(w_rH_) conformational transformations. These reactions are non-dissociative, since they are accompanied by the transformation of the H-bonds and rupture of only some of them. Intermolecular N6H/N6H′…O4/O2 H-bonds exist along all intrinsic reaction coordinate opposite the N3H…N1/N7 H-bonds, that initially weaken and then rupture with a time delay in order to transform into the N3H…N6 H-bond. In other words, in the process of the conformational transformations the N3H group of the T DNA base as proton donor remain for some time free from the intermolecular H-bonding. This comes up with an opinion that discovered conformational transitions could be used for the explanation of the occurrence of the hydrogen-deuterium exchange in the A·T DNA base pairs. It is not excluded that revealed by us novel corridor of the spontaneous thermal fluctuations of the A·T DNA base pairs accompanied by the transformation of the base pair from the plane-symmetric geometry into the significantly non-planar wobble conformation could be useful for the explanation of the specificities of the blurriness of the transition at the DNA pre-melting enriched by the A·T DNA base pairs, that could not be explained in details in the framework of the two-states model.

We would continue to work in the direction of the elucidation of the biological importance of the revealed unusual conformers of the biologically important A·T DNA base pairs.

## Conclusions

In general, in this work at the MP2/aug-cc-pVDZ//B3LYP/6-311++G(d,p) level of theory in the continuum with ε = 4 for the first time we have revealed the A·T(WC)↔A·T(w_WC_), A·T(rWC)↔A·T(w_rWC_), A·T(H)↔A·T(w_H_) and A·T(rH)↔A·T(w_rH_) conformational transformations in the biologically important A·T DNA base pairs and characterized their structural, energetic, polar and dynamical features. These data open new perspectives for the understanding of the physico-chemical mechanisms of the opening of the base pairs preceding DNA melting and also to describe in details the breathing of DNA, that has been experimentally registered. Moreover, it is also the subject for the investigation by using modern spectroscopic techniques such as two-dimensional fluorescent spectroscopy (2DFS) (Widom et al., 2013), time-resolved single molecule fluorescence resonant energy transfer (smFRET) (Lee et al., 2013), single molecule fluorescent linear dichroism (smFLD) (Phelps et al., 2013) and THz spectroscopy (Alexandrov et al., 2013).

## Author contributions

OB, performance of calculations, discussion of the obtained data, preparation of the text of the manuscript. DH, proposition of the task of the investigation, discussion of the obtained data, preparation of the text of the manuscript. KT, preparation of the numerical data for Tables and graphical materials for Figures, preparation of the text of the manuscript. All authors were involved in the proofreading of the final version of the manuscript.

### Conflict of interest statement

The authors declare that the research was conducted in the absence of any commercial or financial relationships that could be construed as a potential conflict of interest.
